# Severe Anxiety and PTSD Symptoms Among Ebola Virus Disease Survivors and Healthcare Workers in the Context of the COVID-19 Pandemic in Eastern DR Congo

**DOI:** 10.3389/fpsyt.2022.767656

**Published:** 2022-05-06

**Authors:** Jude Mary Cénat, Cécile Rousseau, Jacqueline Bukaka, Rose Darly Dalexis, Mireille Guerrier

**Affiliations:** ^1^School of Psychology, University of Ottawa, Ottawa, ON, Canada; ^2^Division of Social and Transcultural Psychiatry, McGill University, Montreal, QC, Canada; ^3^Department of Psychology, University of Kinshasa, Kinshasa, Democratic Republic of Congo; ^4^Interdisciplinary School of Health Sciences, University of Ottawa, Ottawa, ON, Canada

**Keywords:** Ebola virus disease (EVD), COVID-19, anxiety, posttraumatic stress disorder, survivors, healthcare workers (HCWs)

## Abstract

Ebola virus disease (EVD) survivors and healthcare workers (HCWs) face stress, fear, and stigma during the COVID-19 pandemic that can induce severe symptoms of anxiety and post-traumatic stress disorder (PTSD). We examined the prevalence and factors related to severe PTSD and anxiety symptoms, using a representative sample of survivors of the 2018–2020 EVD epidemic in DR Congo in comparison HCWs. Five hundred sixty-three participants (55.25% women, 309 survivors, 202 HCWs, and 52 HCWs and survivors) completed questionnaires assessing anxiety, PTSD, exposure to EVD and COVID-19, stigmatization related to EVD and COVID-19, interpersonal traumas, social support. During the COVID-19 pandemic, 45.6 and 75.0% of survivors and HCWs reported severe symptoms of PTSD and anxiety. Significant difference was observed among the three groups for both PTSD (53.7% survivors, 37.1% HCWs, and 30.8% HCWs-survivors, χ^2^= 18.67, *p* < 0.0001) and anxiety (88.3% survivors, 56.9% HCWs, and 65.4% HCWs- survivors, χ^2^= 67.03, *p* < 0.0001). Comorbidity of severe PTSD and anxiety symptoms was 42.3% between the three groups. Results revealed that exposure to EVD (*b* = 0.53; *p* = 0.001; *b* = 0.12; *p* = 0.042), EVD-related stigmatization (*b* = 0.14; *p* = 0.018; *b* = 0.07; *p* = 0.006), COVID-19-related stigmatization (*b* = 0.22; *p* < 0.0001; *b* = 0.08; *p* = 0.0001) and social support (*b* = −0.30; *p* < 0.0001; *b* = −0.14; *p* < 0.0001) predicted severe PTSD and anxiety symptoms. The last models explained 63.8 and 56.4% of the variance of PTSD and anxiety. Symptoms of PTSD and anxiety are common among EVD survivors and HCWs during the COVID-19 pandemic. Culturally-sensitive programs that address stigma are necessary to mitigate the cumulative effects of EVD and the COVID-19 pandemic on EVD survivors and HCWs.

## Introduction

With four outbreaks occurring from 2018 to 2021, the Democratic Republic of Congo (DRC) faces a constant threat from Ebola virus disease (EVD) (1). The 2018–2020 epidemic in the provinces of North and South Kivu and Ituri in the eastern DRC is the largest the country has faced, and the second largest in the world after the 2013–2016 crisis in West Africa (2, 3). With 2,280 deaths out of 3,463 confirmed and probable cases, despite advances in treatment of EVD over the past 5 years and the most intensive vaccination campaign for EVD, the epidemic has had a mortality rate of 65.83% (3). EVD infected individuals experience acute physical pain and many face isolation, fear, and anxiety because of the death of others in treatment centers (4–9). Health care workers (HCWs), in addition to facing the constant fear of being infected, of infecting family members and loved ones, must also deal with the recurrent death of patients, their distress, and that of their families. During this epidemic, 171 HWCs were infected, representing 5% of cases, and 79 died (3).

The end of this EVD epidemic in the DRC came as the eastern region was affected by the COVID-19 pandemic (10). On October 13, 2020, at the beginning of this study, the DRC counted 10,872 COVID-19 confirmed cases with a low rate of testing (11). This double crisis could reignite both the fear of survivors and HCWs, and the stigmatization to which they have been subjected to according to a process of retraumatization (12). Studies conducted during the first wave of the COVID-19 pandemic in several countries, including the DRC, showed that stigmatization related to the disease was the most significant predictor of mental health problems, including anxiety and depression (13–15). In addition, the eastern region of the DRC has been facing armed conflicts for over 20 years (16). These wars inflicted interpersonal traumas which are a major risk factors for the mental health of EVD survivors and HCWs (17–19). Conducted, in October 2020, in the context of the COVID-19 pandemic, this study aims to: (1) investigate the prevalence of PTSD and anxiety symptoms and their comorbidity among adult EVD survivors and HCWs of the tenth EVD epidemic in the DRC (occurred from August 2018 to June 2020); (2) examine factors related to PTSD and anxiety symptoms and their comorbidity among participants.

## Methods

### Participants and Procedures

We calculated the sample size using Epi Info™. We calculated a population-based sample by considering survivors as the principal group (*n* = 788), and HCWs as the comparison group [*n* = DEFF^*^Np (1-p)]/[(d^2^/${\text{Z}}_{1-\text{α}/2^{*}}^{2}\left(N-1\right)$ +p^*^(1-p)]. Given the population of adult EVD survivors was 788 according to the ministry of health and the Association of Ebola Survivors, with the prevalence of 50% of mental health disorders generally observed (7, 20), a significance level of 5% confidence interval of 95% [50% ± 5 Confidence limits as % of 100 (absolute ± %) (d)], we calculated that the sample size for this study was 259 survivors. For better comparison with HCWs, the same number was considered (n_HCWs_ = 259). Considering a response rate of 70%, the sample size for the study was 740 (370 survivors and 370 HCWs).

Survivors were recruited through the Association of Ebola Survivors (in French: *Association des Vainqueurs d'Ebola*). The Association provided the local coordination of the research team with a contact list (e.g., address, phone number) of individuals willing to be contacted. From this contact list a random sample of 370 survivors was generated by a computer program. HCWs were recruited through the provincial public health departments of North Kivu and Ituri. HCWs' were contacted through the list from the Ebola treatment centers (ETCs) provided by health authorities. The inclusion criteria were: (1) being an EDV survivor or has worked as a HCW (e.g., doctor, nurse) during the epidemic for at least 6 months consecutively; (2) aged 18 years old or more of age; (3) speaks one of the four official languages (Swahili, Lingala, Tshiluba, or Kikongo), French, or English. In total, 746 people were contacted, and 603 agreed to participate. For reasons related to logistics and to travel to areas known to be unsafe, 39 people could not be reached, and three others refused to participate upon contact with interviewer. Of the 746 people contacted, 563 ultimately participated for a response rate of 75.47%. All participants received monetary compensation. Each participant signed a written informed consent. The research ethics committees of the University of Ottawa, the National Institute of Biomedical Research, and the University of Kinshasa approved the study protocol.

Data was collected by 15 interviewers who received two and a half days of training on ethical issues and on how to complete the questionnaire. For the translation and the cultural adaptation of the questionnaire, back-translation methods were used. Translation and cultural adaptation (including cognitive interview) were done by a team of seven Congolese professors, psychologists, and psychiatrists from DRC universities. The investigators were psychologists or individuals with a bachelor's degree in psychology. When first contacted by responsible interviewer, participants also indicated the language in which they wished to be interviewed. All interviewers spoke French and Swahili; some spoke Lingala, Tschiluba, Kinkongo and English. Each province also had a supervisor, who was a psychologist, on site. The survey took place from October 10 to 23, 2020.

A total of 563 participants completed the questionnaire (55.25% women), including 309 survivors (60.26% women), 202 HCWs (51.26% women), and 52 people who were both HCWs and survivors (41.18% women). The mean age of participants was 30.47 years (SD = 10.02). The mean age of survivors was 30.43 years (SD = 10.03), that of HCWs was 29.48 years (SD = 9.49), and that of those who were both survivors and health care workers was 34.57 (SD = 11.09). Table 1 provides details on the sociodemographic characteristics of the survivors.

**Table 1:** Sociodemographic characteristics of the sample over participants' status (*n* = 561).

	**Survivors**	**Healthcare worker**	**Healthcare worker and survivor**	**Total**	***χ***^**2**^, ***p***
Participants' experiences	54.88	35.88	9.24		
**Gender**					
Men	39.74	48.74	58.82	44.75	8.44, 0.015
Women	60.26	51.26	41.18	55.25	
**Age**					
18–24	34.4%	37.4%	10.0%	34.4%	19.60, 0.003
25–34	39.0%	42.6%	58.0%	39.0%	
35–45	17.4%	11.8%	14.0%	17.4%	
45 and more	9.2%	8.2%	18.0%	9.2%	
**Employment status**					
No	62.78	45.05	42.31	54.53	18.94, .001
Yes	37.22	54.95	57.69	45.47	
**Education**					
None	4.21	2.97	3.85	3.73	26.09, < .001
Primary school	12.30	11.88	13.46	12.26	
High school	63.75	46.04	46.15	55.77	
Professional	3.56	6.93	5.77	4.97	
University	16.18	32.18	30.77	23.27	
**Matrimonial status**					
Single	51.14	53.23	27.45	49.73	43.77, < .001
Married	23.13	35.82	50.98	30.23	
Divorced	2.93	2.49	3.92	2.86	
Separated	4.56	4.98	3.92	4.65	
Widowed	15.96	3.48	9.80	10.91	
In a relationship	2.28	0.00	3.92	1.61	
**Positive COVID-19 status**					
No	91.30	88.60	88.50	90.10	1.12; 0.572
Yes	8.70	11.40	11.50	9.90	
**Exposure to EVD**					
Low	5.83	53.96	1.92	22.74	223.15, < .001
Mid	30.42	22.28	7.69	25.40	
High	23.30	20.30	32.69	23.09	
Very High	40.45	3.47	57.69	28.77	
**Stigmatization due to EVD**				
Low	10.68	45.54	23.08	24.33	125.37, < .001
Mid	36.57	20.79	53.85	32.50	
High	13.59	21.78	15.38	16.70	
Very High	39.16	11.88	7.69	26.47	
**Exposure to COVID-19**					
Low	27.83	21.29	9.62	23.80	71.94, < .001
Mid	45.63	23.27	57.69	38.72	
High	12.30	11.39	13.46	12.08	
Very High	14.24	44.06	19.23	25.40	
**Stigmatization due to COVID-19**					
Low	42.07	39.60	61.54	42.98	83.72, < .001
Mid	3.56	13.86	5.77	7.46	
High	14.89	34.16	25.00	22.74	
Very High	39.48	12.38	7.69	26.82	
**Social support**					
Low	38.5%	8.4%	11.5%	25.2%	90.0, < .001
Mid	17.5%	28.2%	46.2%	24.0%	
High	27.5%	25.2%	19.2%	25.9%	
Very High	16.5%	38.1%	23.1%	24.9%	

### Measures

Sociodemographic characteristics assessed included gender, age, employment status, education, and marital status.

#### Degree of Exposure to EVD and COVID-19

An adapted version of the Ebola exposure scale (EES) was used to assess the degree of exposure to both EVD and COVID-19 (13, 21). The items were asked without reference to any disease and participants respond to a column for EVD and another for COVID-19. The ESS is a 17-Yes or No questionnaire that asks about experiences surrounding exposure to the EVD and COVID-19. Precisely, it addresses how EVD affected respondents' lives, their families, and their social networks (e.g., “Have you been in a city or in a village where people became ill because of… EVD/COVID-19?”; “Has a member of your family fallen ill because of … EVD/COVID-19?”; “Have you participated at a funeral of a person deceased because of… EVD/COVID-19?”). The questionnaire is widely used during communities affected by EVD outbreaks and during the COVID-19 pandemic with good internal consistency coefficients (13, 21). In our sample, Cronbach α was 0.84 for EVD and 0.90 for COVID-19.

#### Stigmatization Related to EVD and COVID-19

We used an adapted version of The Stigmatization related to EVD scale which consists of 20 items (14, 21). A 4-point scale evaluates respondents answers: (0) Never, (1) Rarely, (2) Sometimes, (3) Often, and (4) Always. The participants completed two columns for each item: one for stigmatized experience related to EVD (Column 1) and the other for stigmatized experience elated to COVID-19 (“Someone refused to talk to you because of… EVD/COVID-19”; “A company refused to hire you because of… EVD/COVID-19”, etc. This measure was already used in communities affected by EVD outbreaks and during the COVID-19 pandemic with excellent internal consistency (14, 21). In our sample, Cronbach α was 0.98 for EVD and 0.99 for COVID-19.

#### Life Events Checklist for DSM-5

The Life Events Checklist for DSM-5 (LEC-5) is a self-report questionnaire that evaluates traumatic events that have happened in a person's lifetime (22). It consists of 16 specific items that potentially cause distress (e.g., “Sudden accidental death”, “Fire or explosion”) and 1 “other” item in case the first 16 do not capture a person's full experiences. The LEC-5 is composed of a 6-point scale: Happened to me, Witnessed it, Learned about it, Part of my job, Not sure, and Does not apply scored each yes (1) and no (0). In the present study, we used the two first scales Happened to me and Witnessed it with a sum of scores of the items. In our sample, Cronbach α was 0.77 for “Happened to me” and 0.79 for “Witnessed it”.

#### Hopkins Symptom Checklist

We used the Hopkins Symptom Checklist (HSCL) anxiety subscale to assess anxiety symptoms (23). The HSCL anxiety subscale is a 10-item scale. Participants rated each symptom for the last seven days from 1 to 4 (“Not at all”, “A little”, “Quite a bit”, “Extremely”). This subscale is one of the most reliable measures for assessing anxiety symptoms in different cultures (24). It shows excellent internal consistency in studies in different countries (24). It is already used in the DRC with a Cronbach's alpha of 0.91. In our sample, Cronbach's α 0.95. An average score of 1.75 indicates a significant level of anxiety and this was the cutoff point used in our analyses (23).

#### Posttraumatic Stress Disorder Check List for DSM-5

The PTSD Check List for DSM 5 (PCL-5) was used to assess symptoms of Posttraumatic stress disorder (22, 25, 26). The PCL-5 is a 20-item questionnaire that evaluates the 20 symptoms of PTSD found in the DSM-5 (e.g., “In the past month, how much were you been bothered by repeated, disturbing, and unwanted memories of the stressful experience?”). This measure utilizes a 5-point scale: (0) Not at all, (1) A little bit, (2) Moderately, (3) Quite a bit, and (4) Extremely. The PCL-5 has strong internal consistency (α = 0.94), test-retest reliability (r = 0.82), and convergent (rs = 0.74–0.85) and discriminant (rs = 0.31–0.60) validity (25, 27, 28). While this scale is best used by clinicians and can be scored using different methods, it can be most easily scored by summing up the answers for all 20 items (0–80). A cut-off score of 33 suggest severe PTSD symptoms (26). In our sample, the Cronbach's α was 0.97.

#### Multidimensional Scale of Perceived Social Support

The Multidimensional Scale of Perceived Social Support is a 12 item self-report questionnaire (29). It evaluates social support from three sources: family, friends, and significant others (e.g., “There is a special person who is around when I am in need”, “My family really tries to help me”, “I can count on my friends when things go wrong”). It consists of a 6-point scale: (1) Very Strongly Disagree, (2) Strongly Disagree, (3) Mildly Disagree, (4) Neutral, (5) Mildly Agree, (6) Strongly Agree, (7) Very Strongly Agree. The scale showed very good internal consistency with a Cronbach's alpha coefficient of 0.88 (29). In our sample, the Cronbach's α was 0.93.

### Statistical Analyses

Using the Statistical Package for the Social Sciences (SPSS), version 27, we computed the prevalence of severe symptoms of PTSD and anxiety using the above-mentioned cut-off across sociodemographic characteristics including gender, age category, education level, employment status, categories of exposure to EVD and COVID-19, and categories of stigmatization due to EVD and COVID-19. To compare levels of exposure to EVD and COVID-19 and stigmatization related to EVD and COVID-19, their scores were classified in four categories with values below the 25th percentile, between the 25th and 50th percentile, between 50th and 75th, and values beyond the 75th percentile according to past studies (13–15, 20).

Subsequently, a multivariable linear regression was conducted separately to examine the association between mental health symptoms (PTSD and anxiety) and exposure to EVD, exposure to COVID-19, and stigmatization due to EVD and COVID-19. The factors were entered progressively into six different models. First, we tested the sociodemographic characteristics and EVD exposure. Secondly, we added stigmatization due to EVD to the previous list of variables and COVID-19 exposure was then added to the third model. The fourth model included stigmatization due to COVID-19 and the fifth mode included experienced and witnessed interpersonal traumas. Lastly, social support was added in the final model. All the models are presented in Appendix 1 and the final model is presented in the **Table 4**.

We verified the homogeneity of variance using the scatterplot of standardized predicted values vs. the standardized residuals. We tested the normality of the residuals for both symptoms of PTSD and anxiety with the Shapiro-Wilk test and through examination of skewness and kurtosis. Skewness values between −1 and + 1 and kurtosis values between −2 and +2 were deemed acceptable (30, 31). Interaction terms between exposures to the two viruses in conjunction with stigmatization related to EVD and COVID-19 were also tested, but are not presented in the current findings because of non-significant results. Interaction terms were also tested between gender exposure to EVD and COVID-19, stigmatization related to EVD and COVID-19, as well as COVID-19 status. Gender interactions were significant only for exposure to COVID-19 and COVID-19 status in predicting anxiety symptoms. Judged as less pertinent, this ultimate model (Model 7) was presented in the Appendix 1.

## Results

Results presented in the Table 1 showed that 9.9% of the participants declared having tested positive for COVID-19 (8.7% of survivors, 11.4% of HWCs who are also EVD survivors, and 11.5% among those who are HCWs and were not infected by EVD). Generally, 45.6 and 75.0% of the total sample were categorized as having severe symptoms of PTSD and anxiety, respectively. Table 1 outlines statistically significant difference among the three categories of participants for severe symptoms of both PTSD (χ^2^ = 18.67, *p* < 0.0001) and anxiety (χ^2^ = 67.03, *p* < 0.0001). EVD survivors reported higher symptoms of PTSD and anxiety (53.7 and 88.3%, respectively) compared to HCWs (37.1 and 56.9% for symptoms of PTSD and anxiety, respectively) and those who were both survivors and HCWs (30.8 and 65.4% for symptoms of PTSD and anxiety, respectively). Significant difference was also observed between genders, employment status, education level, matrimonial status with higher prevalence observed among women (51.8 vs. 38.9% and 79.9 vs. 70% for symptoms of PTSD and anxiety, respectively), those who are unemployed (59.3 vs. 29.3% and 78.5 vs. 70.7% for symptoms of PTSD and anxiety, respectively) and widowed (77.0 and 93.4% for symptoms of PTSD and anxiety, respectively). Results showed that COVID-19 negative participants reported higher prevalence of anxiety symptoms (76.9%) compared to COVID-19 positive participants (75.1%), χ^2^ = 10.51, *p* < 0.0001. However, there was no significant difference among the two groups for PTSD symptoms. Table 2 presents all results.

**Table 2:** Prevalence of PTSD and anxiety symptoms over participants' status (*n* = 561).

	**PTSD symptoms**					**Anxiety symptoms**				
	**Survivors**	**Healthcare workers**	**Healthcare workers and survivors**	**Total**	***χ***^**2**^, ***p***	**Survivors**	**Healthcare workers**	**Healthcare worker and survivor**	**Total**	***χ***^**2**^, ***p***
Participants' experiences	53.7	37.1	30.8	45.6	18.67; <0.0001	88.3	56.9	65.4	75.0	67.03; <0.0001
**Gender**										
Men	43.3	35.1	33.3	38.9	9.20; 0.002	85.8	51.5	66.7	70.0	6.82; 0.009
Women	61.5	39.2	28.6	51.8		91.2	62.7	61.9	79.7	
**Age**										
18–24	74.3	35.6	40	74.3	20.28; <0.0001	87.6	52.1	40.0	87.6	2.88; 0.411
25–34	47.1	32.5	34.5	47.1		89.1	54.2	69.0	89.1	
35–45	43.4	47.8	42.9	43.4		86.8	60.9	85.7	86.8	
45 and more	28.6	37.5	11.1	28.6		89.3	75.0	66.7	89.3	
**Employment status**										
No	71.1	40.7	31.8	59.3	50.59; <0.0001	90.7	60.4	45.5	78.5	4.52; 0.033
Yes	24.3	34.2	30.0	29.3		84.3	54.1	80.0	70.7	
**Education**										
None	61.5	33.3	50.0	52.4	26.55; <0.0001	100.0	66.7	50.0	85.7	21.22; <0.0001
Primary school	44.7	41.7	14.3	40.6		81.6	58.3	57.1	71.0	
High school	64.5	37.6	25.0	53.5		91.4	61.3	70.8	80.9	
Professional	45.5	50.0	66.7	50.0		81.8	64.3	100.0	75.0	
University	18.0	32.3	37.5	27.5		80.0	47.7	56.3	61.1	
**Matrimonial status**										
Single	53.5	36.4	50.0	46.8	41.39; <0.0001	86.6	57.0	50.0	73.4	14.37; 0.013
Married	31.0	34.7	19.2	30.8		84.5	55.6	73.1	70.4	
Divorced	55.6	40.0	50.0	50.0		77.8	60.0	100.0	75.0	
Separated	78.6	40.0	–	57.7		92.9	50.0	100.0	76.9	
Widowed	81.6	71.4	40.0	77.0		98.0	85.7	60.0	93.4	
In a relationship	57.1	–	50.0	55.6		100.0	–	50.0	88.9	
**Stigmatization due to EVD**										
Low	42.4	28.3	25.0	31.4	229.63; <0.0001	54.5	47.8	16.7	46.7	97.48; <0.0001
Mid	5.3	28.6	14.3	12.0		92.0	64.3	82.1	84.2	
High	64.3	50.0	75.0	58.5		73.8	61.4	75.0	68.1	
Very high	98.3	62.5	75.0	91.9		99.2	70.8	75.0	94.0	
**Positive COVID-19 status**										
No	55	35.2	30.4	45.8	0.03; 0.874	91.5	55.9	69.6	76.9	10.51; 0.001
Yes	40.7	52.2	33.3	44.6		55.6	65.2	33.3	57.1	
**Exposure to COVID-19**										
Low	83.7	32.6	60.0	66.4	46.19; <0.001	88.4	48.8	60.0	74.6	16.80; 0.001
Mid	41.1	17.0	23.3	33.5		93.6	53.2	66.7	81.2	
High	36.8	21.7	14.3	29.4		94.7	56.5	85.7	80.9	
Very high	50.0	53.9	50.0	52.4		65.9	62.9	50.0	62.9	
**Stigmatization due to COVID-19**										
Low	12.3	23.8	6.3	15.3	221.36; <0.0001	90.0	62.5	62.5	77.3	67.27; <0.0001
Mid	54.5	50.0	33.3	50.0		54.5	53.6	33.3	52.4	
High	52.2	37.7	76.9	46.9		63.0	46.4	76.9	55.5	
Very high	98.4	64.0	75.0	92.1		99.2	72.0	75.0	94.0	
**Social support**										
Low	98.3	94.1	83.3	98.3	220.15; <0.0001	98.3	94.1	100.0	98.3	98.01; <0.0001
Mid	53.7	35.1	29.2	53.7		81.5	59.6	58.3	81.5	
High	12.9	25.5	10.0	12.9		92.9	70.6	70.0	92.9	
Very high	17.6	33.8	25.0	17.6		64.7	37.7	58.3	64.7	

Table 3 presents the prevalence of comorbidity of severe PTSD and anxiety symptoms. The results showed a prevalence of comorbidity of 42.3%, with a statistically significant difference between survivors (51.8%), HCWs (31.7%), and survivors and HCWs participants (26.9%; χ^2^ = 63.78, *p* < 0.0001). Similar differences were observed for gender, employment status, education, marital status, stigma related to EVD and COVID-19 and exposure to COVID-19.

**Table 3:** Prevalence of comorbidity of PTSD and anxiety symptoms over participants' status (*n* = 561).

	**Survivors**	**Healthcare workers**	**Healthcare workers and survivors**	**Total**	***χ***^**2**^, ***p***
Participants' experiences	51.8	31.7	26.9	42.3	63.78; <0.0001
**Gender**					
Men	41.7	26.8	26.7	34.0	13.60; 0.001
Women	59.3	36.3	28.6	49.5	
**Age**					
18–24	71.4	34.2	40.0	55.7	47.89; <0.0001
25–34	47.1	25.3	27.6	36.8	
35–45	41.5	39.1	42.9	41.0	
45 and more	21.4	31.3	11.1	22.6	
**Employment status**					
No	69.6	36.3	27.3	56.7	60.85; <0.0001
Yes	21.7	27.9	26.7	25.0	
**Education**					
None	61.5	33.3	50.0	52.4	36.57; <0.0001
Primary school	44.7	37.5	14.3	39.1	
High school	62.9	33.3	16.7	50.6	
Professional	36.4	42.9	66.7	42.9	
University	14.0	24.6	37.5	22.1	
**Matrimonial status**					
Single	52.2	31.8	35.7	43.5	49.19; <0.0001
Married	29.6	27.8	19.2	27.2	
Divorced	44.4	20.0	50.0	37.5	
Separated	71.4	40.0		53.8	
Widowed	79.6	71.4	40.0	75.4	
In a relationship	57.1		50.0	55.6	
**Stigmatization due to EVD**					
Low	30.3	21.7	16.7	23.4	319.41; <0.0001
Mid	5.3	26.2	14.3	11.5	
High	59.5	40.9	62.5	51.1	
Very high	98.3	62.5	75.0	91.9	
**Positive COVID-19 status**					
No	53.9	30.7	26.1	43.2	4.25; 0.119
Yes	29.6	39.1	33.3	33.9	
**Exposure to COVID-19**					
Low	82.6	27.9	60.0	64.2	71.88; <0.0001
Mid	40.4	14.9	20.0	32.1	
High	36.8	17.4	14.3	27.9	
Very high	40.9	46.1	40.0	44.1	
**Stigmatization due to COVID-19**					
Low	10.8	22.5	3.1	13.6	282.46; <0.0001
Mid	45.5	35.7	33.3	38.1	
High	45.7	29.0	69.2	39.1	
Very high	98.4	64.0	75.0	92.1	
**Social support**					
Low	98.3	94.1	83.3	97.2	305.46; <0.0001
Mid	48.1	28.1	25.0	35.6	
High	11.8	21.6	10.0	15.1	
Very high	13.7	27.3	16.7	21.4	

As depicted in Appendix 1, we separately estimated a series of six models to predict symptoms of PTSD and anxiety by successively adding stigmatization due to EVD, exposure to COVID-19, stigmatization due to COVID-19, interpersonal traumas, and social support to the last model which comprised sociodemographic characteristics and exposure to EVD. All six models predicting symptoms of PTSD were significant with explained variance percentages ranging from 27.7 to 63.8%, with 63.8% of variance explained for the final model [*F*_(18, 516)_ = 50.43; *p* < 0.0001]. Results showed that EVD exposure (*b* = 0.53; *p* = 0.001), stigmatization due to EVD (*b* = 0.14; *p* = 0.018), stigmatization due to COVID-19 (*b* = 0.22; *p* < 0.0001) and social support (*b* = −0.30; *p* < 0.0001) predicted PTSD symptoms, whereas exposure to COVID-19 and exposure and witnessing of traumatic events did not (*b* = 0.14; *p* = 0.35; *b* = −0.02; *p* = 0.49; *b* = 0.04; *p* = 0.21). Similarly, the models for symptoms of anxiety were all significant with explained variance percentages between 26.4 and 56.4%. The final model [*F*_(18, 516)_ = 37.14; *p* < 0.0001] presented in Table 4 indicated that exposure to EVD (*b* = 0.12; *p* = 0.042), stigmatization due to EVD (*b* = 0.07; *p* = 0.006), and social support (*b* = −0.14; *p* < 0.0001) predicted symptoms of anxiety. Stigmatization related to COVID-19 significantly predicted anxiety symptoms until the 5th model that did not include social support (*b* = 0.08; *p* = 0.0001).

**Table 4:** Results of linear regression analyses predicting symptoms of PTSD and anxiety.

	**Coefficients**					**Coefficients**				
	**B**	**Beta**	***P*** **-value**	**95.0% CI**		**B**	**Beta**	***P*** **-value**	**95.0% CI**	
	**PTSD symptoms**					**Anxiety symptoms**				
	***Model 6: F***_**(18, 516)**_ **= 50.43**, ***p*** **< 0.0001; R2 = 63.8**					***F***_**(18, 516)**_ **= 37.14**, ***p*** **< 0.0001; R2 = 56.4**				
Gender	1.19	0.03	0.233	−0.769	3.146	0.60	0.05	0.128	−0.17	1.38
Age	−0.03	−0.02	0.665	−0.148	0.095	−0.02	−0.03	0.392	−0.07	0.03
Employment Status	−0.68	−0.02	0.546	−2.88	1.524	−0.32	−0.02	0.475	−1.19	0.55
**Education**										
None	6.66	0.07	0.019	1.092	12.218	2.36	0.07	0.036	0.16	4.56
Primary school	0.36	0.01	0.841	−3.136	3.851	0.48	0.02	0.498	−0.91	1.86
High school	2.80	0.08	0.027	0.326	5.277	1.38	0.11	0.006	0.40	2.36
Professional	2.96	0.04	0.213	−1.7	7.611	3.09	0.11	0.001	1.25	4.93
**Matrimonial status**										
Married	0.31	0.01	0.808	−2.205	2.826	−0.12	−0.01	0.816	−1.11	0.88
Divorced	1.85	0.04	0.226	−1.147	4.848	0.24	0.02	0.686	−0.94	1.43
**Participants' experiences**										
Healthcare worker	−2.39	−0.06	0.089	−5.15	0.367	−0.81	−0.06	0.145	−1.90	0.28
Healthcare worker and survivor	−3.05	−0.05	0.087	−6.541	0.442	−1.36	−0.06	0.054	−2.74	0.02
Exposure to EVD	0.53	0.12	0.001	0.206	0.861	0.12	0.08	0.042	−0.01	0.25
Stigmatization due to EVD	0.14	0.18	0.018	0.025	0.262	0.07	0.22	0.006	0.02	0.11
Exposure to COVID-19	0.14	0.03	0.345	−0.152	0.433	0.01	0.00	0.928	−0.11	0.12
Stigmatization due to COVID-19	0.22	0.31	<0.0001	0.104	0.327	0.02	0.09	0.306	−0.02	0.07
Exposure to traumatic events	−0.16	−0.02	0.494	−0.635	0.307	0.06	0.02	0.517	−0.13	0.25
Witness of traumatic events	0.26	0.04	0.205	−0.142	0.662	−0.04	−0.02	0.617	−0.20	0.12
Social support	−0.30	−0.32	<0.0001	−0.375	−0.224	−0.14	−0.41	<0.0001	−0.17	−0.11

*Reference categories are the following: Sex: Men; Employment status: unemployed; Education level: university; Marital status: Single; participants ‘experiences: survivors; B: Unstandardized coefficients; Beta: standardized coefficients*.

*To read the five first models, please, see Appendix 1*.

## Discussion

This is one of the first studies on the mental health of survivors and HCWs during the second-largest EVD epidemic in these particular conflict-affected regions. However, it is the first which also investigates the impact of the COVID-19 pandemic on the mental health of groups most affected by EVD. It is also one of the largest studies to have assessed PTSD and anxiety symptoms in both EVD survivors and HCWs. Studies conducted on anxiety and PTSD following the 2013–2016 crisis in West Africa among survivors have included 8–268 participants (32–37), except a recent large study (*n* = 1,495) conducted in Liberia, Guinea, and Sierra Leone that explored depression, and anxiety symptoms, but not PTSD (38). The results of the present study showed that symptoms of PTSD and anxiety are common among EVD survivors and HCWs during the COVID-19 pandemic. Nearly one out of two participants (45.6%) and three out of four (75%) had severe symptoms of PTSD and anxiety. Participants who were only survivors were more likely to experience severe symptoms of both PTSD and anxiety, respectively, more than half (53.7%) and nearly 9 out of 10 survivors (88.3%). When we observe the two other groups, results also showed that HCWs participants were more likely to experience severe symptoms of PTSD compared to Survivors HCWs. However, those who were both HCWs and survivors were more likely to experience severe symptoms of anxiety during the COVD-19 pandemic compared to HCWs who were not infected by the EVD. Although not a longitudinal design, the very high rates of PTSD and anxiety symptoms reported by survivors and HCWs may suggest that the COVID-19 pandemic may have increased or reactivated the trauma-related symptoms associated with having experienced EVD. The few studies conducted among survivors and HCWs all found lower prevalence of PTSD (39, 40). A study among 68 survivors in Guinea who visited a psychiatric hospital found that <10% had severe symptoms of PTSD (39). Another study among aid workers including HCWs found that up to 40% of some participants showed symptoms of PTSD (40). However, a recent study conducted in the DRC among a representative sample of urban and rural areas affected by EVD in the Equateur Province showed that 58.81% of participants experienced severe symptoms of PTSD. The studies conducted to date on anxiety symptoms in the context of EVD showed a prevalence varying between 19.2 and 83.3% (38, 41). A study of 8 survivors in the United States showed that 6 of them (75%) had significant symptoms of anxiety (32), while another study involving 18 survivors in Sierra Leone found a prevalence of 83.3% of anxiety symptoms (34). Results from the present study reveal the highest prevalence of severe anxiety symptoms both among survivors and HCWs compared to other studies (38, 41). This study also showed greater anxiety symptoms among HCWs in eastern DRC compared to other studies conducted after the 2013–2016 epidemic (34).

This study has also demonstrated significant gender differences, with women showing more anxiety and PTSD symptoms than men. While this is often observed in studies on anxiety and PTSD, these differences are worth noting since studies conducted in the context of both EVD and COVID-19 in the DRC, Togo and Rwanda have shown that there were no such differences (13, 14, 21). The results further indicated that EVD and COVID-19-related stigma were related to both anxiety and PTSD. First, the results showed that participants with a higher stigma score were more likely to have met criteria for severe anxiety and PTSD symptoms during the COVID-19 pandemic. Second, the results of the different regression models also showed that EVD-related stigma experienced by survivors and HCWs was the variable that best and consistently predicted both severe anxiety and PTSD symptoms. This appears consistent with studies where EDV-related stigma plays an important role in mental health (21, 37). Additionally, adding EVD-related stigma to the second models increased the level of explained variance from 27.7 to 56.1% for PTSD symptoms and from 26.4 to 47.6% for anxiety symptoms. Third, the results showed that COVID-19 stigma was also a strong predictor of anxiety and PTSD symptoms. Studies among populations affected by the COVID-19 pandemic and HCWs have shown a positive association between COVID-19-related stigma and anxiety, PTSD and other mental health problems (13, 42, 43). These results indicate that despite the education campaigns for both EVD and COVID-19, people continue to experience stigma related to these infectious diseases (44), which has a significant impact on mental health. These results could be explained by the violent nature of the stigmatization experienced as a result of both illnesses, the lack of social support and the loss of belonging, but also since victimization is often long-term (4, 45–48). Again, without demonstrating causality, results suggest that the COVID-19 pandemic may reactivate the suffering of survivors and HCWs related to stigma, and the fear of catching the disease and being re-victimized.

Although the results showed significant differences by level of exposure to COVID-19, they did not predict anxiety and PTSD symptoms. Social support buffered the effect of COVID-19-related stigma. Indeed, despite all of the adversity experienced, social support was found to be a protective factor for anxiety and PTSD symptoms, which is in keeping with studies on collective trauma (49–51).

### Limitations

Although this study addresses an important issue in depth, it has limitations. First, a longitudinal design would have facilitated the study of causal factors related to severe anxiety symptoms and PTSD. It would also have allowed for an examination of the trajectory of anxiety and PTSD symptoms and stigma experiences, but also whether the onset of the COVID-19 pandemic was instrumental in increasing symptoms. Second, it would have been relevant to assess symptoms before and after both EVD and COVID-19. Although this was considered, after discussion with Congolese colleagues, this perspective was forgotten given the trouble it could cause participants to assess symptoms experienced during multiple traumatic periods and this would increase the risk of recall bias (EVD and COVID-19-related stigma, and interpersonal trauma). Third, the status of health care workers (e.g., front line, second line) which was not evaluated constitute a limitation since studies on COVID-19 showed differences in mental health issues related to the pandemic (52, 53). Although only health care professionals who worked in ETCs were included and the well-known high level of contagiousness of EVD, assessment of the health care professional's status would have allowed analysis of the presence of differences depending on whether the participant worked in the front line or was in direct contact with patients. Finally, it is also important to note that the traumatic events experienced do not predict symptoms of anxiety and PTSD. However, the questionnaire assessing traumatic events, the LEC-5, was very broad and assessed different types of events ranging from interpersonal to non-interpersonal traumas. Future studies should use questionnaires that are more focused and accurately assess interpersonal trauma related to the current unstable security situation in the eastern DRC region (54). There is a specific measure of the experiences of people living in the eastern DRC region. This may better account for the impacts on PTSD and anxiety symptoms of these major traumatic experiences where people are killed, where rape is a weapon of war (55).

### Conclusions

Despite these limitations, the results offer unique insights into the possible cumulative effects of consecutive serious epidemics and have important implications for research, public health policy, and clinical practice. First, given the high proportion of variance of anxiety and PTSD symptoms explained by the variables investigated and the predictive role of the experience of stigma related to both EVD and COVID-19, innovative health communications strategies to minimize stigma related to health crises and its harmful effect on social fabric and on individual mental health are required. Second, this study shows the importance of providing long-term care for survivors and HCWs. The results indicate that these follow-ups, in addition to addressing physical and mental health issues, should also address social, community, and anthropological factors (e.g., cultural perspectives of health, health communication and practices, economic and education aspects, social organization and values) to provide adequate and culturally appropriate care for survivors and HCWs, but also other affected groups such as orphans, widows and widowers, and families. Finally, longitudinal studies are needed to document the medium-and long-term effects of health crises on those recovered. The World Health Organization could support low-and-middle income countries' public health structures in this regard and ensure an open database that could play an important role in the development of public health policies and education and communication campaigns on infectious diseases. This study also shows the need to develop qualitative and ethnographic studies that can deeply explore the traumatic experiences of survivors and healthcare workers, the stigma they face, their daily lives as survivors and health care workers, their social status, gender roles, and the social and economic losses caused by the Ebola outbreak. By addressing these issues, the studies will help in the development of mental health programs that meet the real needs of survivors, health professionals and the communities.

## Data Availability Statement

The raw data supporting the conclusions of this article will be made available by the authors, without undue reservation.

## Ethics Statement

The studies involving human participants were reviewed and approved by University of Ottawa Research Ethics Board and the University of Kinshasa. The patients/participants provided their written informed consent to participate in this study.

## Author Contributions

JMC, RD, CR, JB, and MG: conceptualization. JMC, CR, and JB: investigation and acquisition of data and writing—review and editing. MG and JMC: software and formal analysis. JMC, MG, RD, and CR: interpretation of data. JMC, MG, and RD: writing—original draft. All authors contributed to the article and approved the submitted version.

## Funding

This article was supported by the grant #108968 from the International Development Research Centre (IDRC), in collaboration with the Social Sciences and Humanities Research Council (SSHRC), and the Canadian Institutes of Health Research (CIHR).

## Conflict of Interest

The authors declare that the research was conducted in the absence of any commercial or financial relationships that could be construed as a potential conflict of interest.

## Publisher's Note

All claims expressed in this article are solely those of the authors and do not necessarily represent those of their affiliated organizations, or those of the publisher, the editors and the reviewers. Any product that may be evaluated in this article, or claim that may be made by its manufacturer, is not guaranteed or endorsed by the publisher.
