# Supplementary material for: Severe Anxiety and PTSD Symptoms Among Ebola Virus Disease Survivors and Healthcare Workers in the Context of the COVID-19 Pandemic in Eastern DR Congo
Source: Front Psychiatry. 2022 May 6;13:767656. doi: 10.3389/fpsyt.2022.767656 (PMC9120641; doi:10.3389/fpsyt.2022.767656)
Supplement: Supplementary file 1 [file Table_1.DOCX]

Appendix 1. Results of the six Linear Regression Models Predicting Symptoms of PTSD and Anxiety

|  | Coefficients | |  |  |  |  | Coefficients | |  |  |  |
| --- | --- | --- | --- | --- | --- | --- | --- | --- | --- | --- | --- |
|  | B | Beta | P-Value | 95.0% CI | |  | B | Beta | P-Value | 95.0% CI | |
|  | PTSD Symptoms | | | | |  | Anxiety Symptoms | | | | |
| Model 1: F (12, 522) = 16.65, *p* <0.0001; R2= 27.7 | | | | | |  | F (12, 522) = 15.62, *p* <0.0001; R2= 26.4 | | | | |
| Gender | 1.08 | 0.03 | 0.438 | -1.66 | 3.82 |  | 0.58 | 0.05 | 0.252 | -0.42 | 1.58 |
| Age | -0.28 | -0.15 | 0.001 | -0.44 | -0.11 |  | -0.09 | -0.13 | 0.006 | -0.15 | -0.03 |
| Employment Status | -6.13 | -0.17 | <.0001 | -9.11 | -3.14 |  | -2.25 | -0.17 | <.0001 | -3.34 | -1.16 |
| Education |  |  |  |  |  |  |  |  |  |  |  |
| None | 11.86 | 0.13 | 0.003 | 4.12 | 19.59 |  | 3.65 | 0.11 | 0.011 | 0.83 | 6.46 |
| Primary school | 3.75 | 0.07 | 0.130 | -1.11 | 8.60 |  | 1.55 | 0.08 | 0.086 | -0.22 | 3.32 |
| High school | 7.34 | 0.20 | <.0001 | 3.97 | 10.72 |  | 2.76 | 0.21 | <.0001 | 1.53 | 3.98 |
| Professional | 5.48 | 0.07 | 0.096 | -0.98 | 11.95 |  | 4.24 | 0.15 | <.0001 | 1.88 | 6.59 |
| Matrimonial Status |  |  |  |  |  |  |  |  |  |  |  |
| Married | 1.44 | 0.04 | 0.422 | -2.08 | 4.96 |  | 0.15 | 0.01 | 0.818 | -1.13 | 1.43 |
| Divorced | 8.52 | 0.19 | <.0001 | 4.44 | 12.61 |  | 2.29 | 0.14 | 0.003 | 0.80 | 3.77 |
| Participants' experiences |  |  |  |  |  |  |  |  |  |  |  |
| Healthcare Worker | -11.99 | -0.32 | <.0001 | -15.59 | -8.39 |  | -4.26 | -0.32 | <.0001 | -5.57 | -2.95 |
| Healthcare Worker & Survivor | -6.33 | -0.10 | 0.011 | -11.18 | -1.49 |  | -2.35 | -0.10 | <.0001 | -4.11 | -0.58 |
| Exposure to Ebola | -0.24 | -0.05 | 0.266 | -0.65 | 0.18 |  | -0.07 | -0.05 | 0.344 | -0.23 | 0.08 |
| Model 2: F (13, 521) = 51.16, *p* <0.0001; R2= 56.1 | | | | | |  | F (13, 521) = 36.39, *p* <0.0001; R2= 47.6 | | | | |
| Gender | 0.92 | 0.03 | 0.398 | -1.22 | 3.06 |  | 0.53 | 0.04 | 0.216 | -0.31 | 1.38 |
| Age | -0.09 | -0.05 | 0.172 | -0.22 | 0.04 |  | -0.03 | -0.04 | 0.31 | -0.08 | 0.03 |
| Employment Status | -2.11 | -0.06 | 0.08 | -4.48 | 0.25 |  | -1.00 | -0.08 | 0.036 | -1.93 | -0.07 |
| Education |  |  |  |  |  |  |  |  |  |  |  |
| None | 6.62 | 0.07 | 0.032 | 0.55 | 12.68 |  | 2.01 | 0.06 | 0.099 | -0.38 | 4.40 |
| Primary school | 2.45 | 0.04 | 0.205 | -1.34 | 6.23 |  | 1.14 | 0.06 | 0.133 | -0.35 | 2.64 |
| High school | 4.83 | 0.13 | <.0001 | 2.18 | 7.48 |  | 1.97 | 0.15 | <.0001 | 0.93 | 3.02 |
| Professional | 3.62 | 0.05 | 0.159 | -1.43 | 8.67 |  | 3.66 | 0.13 | <.0001 | 1.67 | 5.65 |
| Matrimonial Status |  |  |  |  |  |  |  |  |  |  |  |
| Married | 0.75 | 0.02 | 0.591 | -1.99 | 3.50 |  | -0.06 | -0.01 | 0.907 | -1.15 | 1.02 |
| Divorced | 4.24 | 0.09 | 0.01 | 1.02 | 7.46 |  | 0.95 | 0.06 | 0.143 | -0.32 | 2.22 |
| Participants' experiences |  |  |  |  |  |  |  |  |  |  |  |
| Healthcare Worker | -4.14 | -0.11 | 0.006 | -7.06 | -1.21 |  | -1.81 | -0.13 | 0.002 | -2.97 | -0.66 |
| Healthcare Worker & Survivor | -1.94 | -0.03 | 0.318 | -5.75 | 1.87 |  | -0.98 | -0.04 | 0.202 | -2.48 | 0.52 |
| Exposure to Ebola | 0.05 | 0.01 | 0.767 | -0.28 | 0.38 |  | 0.02 | 0.01 | 0.809 | -0.11 | 0.15 |
| Stigmatization due to Ebola | 0.50 | 0.61 | <.0001 | 0.45 | 0.56 |  | 0.16 | 0.53 | <.0001 | 0.14 | 0.18 |
| Model 3: F (14, 520) = 47.42, *p* <0.0001; R2= 56.1 | | | | | |  | F (14, 520) = 34.24, *p* <0.0001; R2= 48.0 | | | | |
| Gender | 0.92 | 0.03 | 0.399 | -1.22 | 3.06 |  | 0.54 | 0.04 | 0.207 | -0.30 | 1.38 |
| Age | -0.09 | -0.05 | 0.175 | -0.22 | 0.04 |  | -0.03 | -0.05 | 0.243 | -0.08 | 0.02 |
| Employment Status | -2.12 | -0.06 | 0.082 | -4.52 | 0.27 |  | -0.87 | -0.07 | 0.071 | -1.81 | 0.08 |
| Education |  |  |  |  |  |  |  |  |  |  |  |
| None | 6.61 | 0.07 | 0.033 | 0.54 | 12.68 |  | 2.11 | 0.06 | 0.083 | -0.28 | 4.49 |
| Primary school | 2.44 | 0.04 | 0.207 | -1.36 | 6.24 |  | 1.20 | 0.06 | 0.114 | -0.29 | 2.69 |
| High school | 4.82 | 0.13 | <.0001 | 2.16 | 7.48 |  | 2.06 | 0.16 | <.0001 | 1.01 | 3.10 |
| Professional | 3.63 | 0.05 | 0.159 | -1.43 | 8.68 |  | 3.60 | 0.12 | <.0001 | 1.61 | 5.59 |
| Matrimonial Status |  |  |  |  |  |  |  |  |  |  |  |
| Married | 0.75 | 0.02 | 0.592 | -2.00 | 3.50 |  | -0.04 | 0.00 | 0.941 | -1.12 | 1.04 |
| Divorced | 4.23 | 0.09 | 0.01 | 1.00 | 7.46 |  | 1.02 | 0.06 | 0.115 | -0.25 | 2.29 |
| Participants' experiences |  |  |  |  |  |  |  |  |  |  |  |
| Healthcare Worker | -4.15 | -0.11 | 0.007 | -7.15 | -1.16 |  | -1.58 | -0.12 | 0.009 | -2.75 | -0.40 |
| Healthcare Worker & Survivor | -1.94 | -0.03 | 0.318 | -5.76 | 1.88 |  | -0.91 | -0.04 | 0.233 | -2.41 | 0.59 |
| Exposure to Ebola | 0.05 | 0.01 | 0.767 | -0.28 | 0.38 |  | 0.02 | 0.01 | 0.804 | -0.11 | 0.15 |
| Stigmatization due to Ebola | 0.50 | 0.61 | <.0001 | 0.45 | 0.56 |  | 0.16 | 0.53 | <.0001 | 0.13 | 0.18 |
| Exposure to COVID-19 | 0.01 | 0.00 | 0.955 | -0.29 | 0.31 |  | -0.12 | -0.07 | 0.052 | -0.24 | 0.00 |
| Model 4: F (15, 519) = 50.75, *p* <0.0001; R2= 59.5 | | | | | |  | F (15, 519) = 33.48, *p* <0.0001; R2= 49.2 | | | | |
| Gender | 1.24 | 0.03 | 0.238 | -0.82 | 3.30 |  | 0.61 | 0.05 | 0.151 | -0.22 | 1.44 |
| Age | -0.01 | -0.01 | 0.894 | -0.14 | 0.12 |  | -0.01 | -0.02 | 0.623 | -0.07 | 0.04 |
| Employment Status | -1.49 | -0.04 | 0.204 | -3.80 | 0.82 |  | -0.73 | -0.06 | 0.126 | -1.66 | 0.21 |
| Education |  |  |  |  |  |  |  |  |  |  |  |
| None | 6.53 | 0.07 | 0.028 | 0.70 | 12.37 |  | 2.09 | 0.06 | 0.082 | -0.27 | 4.45 |
| Primary school | 1.89 | 0.03 | 0.31 | -1.76 | 5.54 |  | 1.08 | 0.05 | 0.15 | -0.39 | 2.56 |
| High school | 4.24 | 0.12 | 0.001 | 1.67 | 6.80 |  | 1.93 | 0.15 | <.0001 | 0.89 | 2.97 |
| Professional | 4.08 | 0.05 | 0.1 | -0.78 | 8.95 |  | 3.70 | 0.13 | <.0001 | 1.73 | 5.66 |
| Matrimonial Status |  |  |  |  |  |  |  |  |  |  |  |
| Married | 0.11 | 0.00 | 0.937 | -2.54 | 2.76 |  | -0.18 | -0.01 | 0.742 | -1.25 | 0.89 |
| Divorced | 2.56 | 0.06 | 0.11 | -0.58 | 5.71 |  | 0.66 | 0.04 | 0.308 | -0.61 | 1.93 |
| Participants' experiences |  |  |  |  |  |  |  |  |  |  |  |
| Healthcare Worker | -3.61 | -0.10 | 0.014 | -6.49 | -0.72 |  | -1.46 | -0.11 | 0.014 | -2.62 | -0.29 |
| Healthcare Worker & Survivor | -2.36 | -0.04 | 0.208 | -6.03 | 1.32 |  | -1.00 | -0.04 | 0.186 | -2.49 | 0.48 |
| Exposure to Ebola | 0.48 | 0.11 | 0.006 | 0.14 | 0.82 |  | 0.11 | 0.07 | 0.118 | -0.03 | 0.25 |
| Stigmatization due to Ebola | 0.14 | 0.17 | 0.022 | 0.02 | 0.26 |  | 0.08 | 0.26 | 0.002 | 0.03 | 0.13 |
| Exposure to COVID-19 | -0.17 | -0.03 | 0.261 | -0.46 | 0.13 |  | -0.16 | -0.09 | 0.01 | -0.27 | -0.04 |
| Stigmatization due to COVID-19 | 0.35 | 0.51 | <.0001 | 0.25 | 0.46 |  | 0.08 | 0.30 | <.0001 | 0.03 | 0.12 |
| Model 5: F (17, 517) = 44.73, *p* <0.0001; R2= 59.5 | | | | | |  | F (17, 517) = 29.55, *p* <0.0001; R2= 49.3 | | | | |
| Gender | 1.22 | 0.03 | 0.249 | -0.851 | 3.281 |  | 0.61 | 0.05 | 0.15 | -0.22 | 1.45 |
| Age | -0.01 | -0.01 | 0.887 | -0.137 | 0.119 |  | -0.01 | -0.02 | 0.629 | -0.06 | 0.04 |
| Employment Status | -1.52 | -0.04 | 0.197 | -3.835 | 0.793 |  | -0.71 | -0.06 | 0.135 | -1.65 | 0.22 |
| Education |  |  |  |  |  |  |  |  |  |  |  |
| None | 6.32 | 0.07 | 0.035 | 0.449 | 12.194 |  | 2.20 | 0.07 | 0.069 | -0.17 | 4.58 |
| Primary school | 1.76 | 0.03 | 0.347 | -1.911 | 5.428 |  | 1.14 | 0.06 | 0.133 | -0.35 | 2.62 |
| High school | 4.12 | 0.11 | 0.002 | 1.526 | 6.705 |  | 2.00 | 0.15 | <.0001 | 0.95 | 3.04 |
| Professional | 4.21 | 0.05 | 0.093 | -0.697 | 9.11 |  | 3.68 | 0.13 | <.0001 | 1.70 | 5.66 |
| Matrimonial Status |  |  |  |  |  |  |  |  |  |  |  |
| Married | 0.14 | 0.00 | 0.92 | -2.52 | 2.792 |  | -0.20 | -0.01 | 0.714 | -1.27 | 0.87 |
| Divorced | 2.64 | 0.06 | 0.101 | -0.517 | 5.798 |  | 0.62 | 0.04 | 0.344 | -0.66 | 1.89 |
| Participants' experiences |  |  |  |  |  |  |  |  |  |  |  |
| Healthcare Worker | -3.69 | -0.10 | 0.012 | -6.583 | -0.8 |  | -1.42 | -0.11 | 0.017 | -2.59 | -0.25 |
| Healthcare Worker & Survivor | -2.33 | -0.04 | 0.214 | -6.011 | 1.352 |  | -1.02 | -0.05 | 0.178 | -2.51 | 0.47 |
| Exposure to Ebola | 0.50 | 0.11 | 0.005 | 0.156 | 0.847 |  | 0.10 | 0.07 | 0.144 | -0.04 | 0.24 |
| Stigmatization due to Ebola | 0.16 | 0.19 | 0.015 | 0.03 | 0.28 |  | 0.07 | 0.24 | 0.005 | 0.02 | 0.12 |
| Exposure to COVID-19 | -0.18 | -0.04 | 0.223 | -0.48 | 0.112 |  | -0.15 | -0.08 | 0.016 | -0.27 | -0.03 |
| Stigmatization due to COVID-19 | 0.34 | 0.49 | <.0001 | 0.226 | 0.451 |  | 0.08 | 0.32 | 0.001 | 0.04 | 0.13 |
| Exposure to traumatic events | -0.18 | -0.03 | 0.482 | -0.675 | 0.319 |  | 0.06 | 0.02 | 0.591 | -0.15 | 0.26 |
| Witness of traumatic events | 0.16 | 0.02 | 0.451 | -0.26 | 0.59 |  | -0.09 | -0.03 | 0.323 | -0.26 | 0.09 |
| Model 6: F (18, 516) = 50.43, *p* <0.0001; R2= 63.8 | | | | | |  | F (18, 516) = 37.14, *p* <0.0001; R2= 56.4 | | | | |
| Gender | 1.19 | 0.03 | 0.233 | -0.769 | 3.146 |  | 0.60 | 0.05 | 0.128 | -0.17 | 1.38 |
| Age | -0.03 | -0.02 | 0.665 | -0.148 | 0.095 |  | -0.02 | -0.03 | 0.392 | -0.07 | 0.03 |
| Employment Status | -0.68 | -0.02 | 0.546 | -2.88 | 1.524 |  | -0.32 | -0.02 | 0.475 | -1.19 | 0.55 |
| Education |  |  |  |  |  |  |  |  |  |  |  |
| None | 6.66 | 0.07 | 0.019 | 1.092 | 12.218 |  | 2.36 | 0.07 | 0.036 | 0.16 | 4.56 |
| Primary school | 0.36 | 0.01 | 0.841 | -3.136 | 3.851 |  | 0.48 | 0.02 | 0.498 | -0.91 | 1.86 |
| High school | 2.80 | 0.08 | 0.027 | 0.326 | 5.277 |  | 1.38 | 0.11 | 0.006 | 0.40 | 2.36 |
| Professional | 2.96 | 0.04 | 0.213 | -1.7 | 7.611 |  | 3.09 | 0.11 | 0.001 | 1.25 | 4.93 |
| Matrimonial Status |  |  |  |  |  |  |  |  |  |  |  |
| Married | 0.31 | 0.01 | 0.808 | -2.205 | 2.826 |  | -0.12 | -0.01 | 0.816 | -1.11 | 0.88 |
| Divorced | 1.85 | 0.04 | 0.226 | -1.147 | 4.848 |  | 0.24 | 0.02 | 0.686 | -0.94 | 1.43 |
| Participants' experiences |  |  |  |  |  |  |  |  |  |  |  |
| Healthcare Worker | -2.39 | -0.06 | 0.089 | -5.15 | 0.367 |  | -0.81 | -0.06 | 0.145 | -1.90 | 0.28 |
| Healthcare Worker & Survivor | -3.05 | -0.05 | 0.087 | -6.541 | 0.442 |  | -1.36 | -0.06 | 0.054 | -2.74 | 0.02 |
| Exposure to Ebola | 0.53 | 0.12 | 0.001 | 0.206 | 0.861 |  | 0.12 | 0.08 | 0.042 | -0.01 | 0.25 |
| Stigmatization due to Ebola | 0.14 | 0.18 | 0.018 | 0.025 | 0.262 |  | 0.07 | 0.22 | 0.006 | 0.02 | 0.11 |
| Exposure to COVID-19 | 0.14 | 0.03 | 0.345 | -0.152 | 0.433 |  | 0.01 | 0.00 | 0.928 | -0.11 | 0.12 |
| Stigmatization due to COVID-19 | 0.22 | 0.31 | <.0001 | 0.104 | 0.327 |  | 0.02 | 0.09 | 0.306 | -0.02 | 0.07 |
| Exposure to traumatic events | -0.16 | -0.02 | 0.494 | -0.635 | 0.307 |  | 0.06 | 0.02 | 0.517 | -0.13 | 0.25 |
| Witness of traumatic events | 0.26 | 0.04 | 0.205 | -0.142 | 0.662 |  | -0.04 | -0.02 | 0.617 | -0.20 | 0.12 |
| Social Support | -0.30 | -0.32 | <.0001 | -0.375 | -0.224 |  | -0.14 | -0.41 | <.0001 | -0.17 | -0.11 |
|  | Model 7: F (24, 510) = 39.91, *p* <0.0001; R2= 65.3 | | | | |  | F (24, 510) = 29.83, *p* <0.0001; R2= 58.4 | | | | |
| Gender | 6.481 | 0.18 | 0.027 | 0.755 | 12.207 |  | 4.294 | 0.33 | <.001 | 2.032 | 6.556 |
| Age | -0.003 | -0.001 | 0.967 | -0.123 | 0.118 |  | -0.013 | -0.019 | 0.603 | -0.06 | 0.035 |
| Employment Status | -0.722 | -0.02 | 0.519 | -2.921 | 1.477 |  | -0.313 | -0.024 | 0.479 | -1.182 | 0.555 |
| Education |  |  |  |  |  |  |  |  |  |  |  |
| None | 3.265 | 0.035 | 0.223 | -1.992 | 8.522 |  | 0.769 | 0.023 | 0.467 | -1.308 | 2.845 |
| Primary school | -2.114 | -0.038 | 0.176 | -5.178 | 0.949 |  | -0.848 | -0.042 | 0.169 | -2.058 | 0.362 |
| High school | -0.033 | 0 | 0.988 | -4.431 | 4.366 |  | 1.698 | 0.059 | 0.055 | -0.039 | 3.435 |
| Professional | -2.897 | -0.068 | 0.021 | -5.36 | -0.435 |  | -1.557 | -0.101 | 0.002 | -2.53 | -0.585 |
| Matrimonial Status |  |  |  |  |  |  |  |  |  |  |  |
| Married | 0.027 | 0.001 | 0.983 | -2.459 | 2.512 |  | 0.27 | 0.021 | 0.589 | -0.711 | 1.252 |
| Divorced | 1.623 | 0.036 | 0.272 | -1.274 | 4.521 |  | 0.479 | 0.029 | 0.411 | -0.665 | 1.624 |
| Participants' experiences |  |  |  |  |  |  |  |  |  |  |  |
| Healthcare Worker | -3.928 | -0.105 | 0.008 | -6.811 | -1.044 |  | -1.085 | -0.08 | 0.062 | -2.223 | 0.054 |
| Healthcare Worker & Survivor | -3.042 | -0.049 | 0.085 | -6.511 | 0.426 |  | -1.444 | -0.064 | 0.039 | -2.814 | -0.074 |
| Exposure to Ebola | 0.571 | 0.129 | 0.018 | 0.1 | 1.041 |  | 0.259 | 0.162 | 0.006 | 0.073 | 0.444 |
| Stigmatization due to Ebola | 0.176 | 0.215 | 0.044 | 0.005 | 0.347 |  | 0.074 | 0.249 | 0.032 | 0.006 | 0.142 |
| Exposure to COVID-19 | 0.647 | 0.124 | 0.02 | 0.103 | 1.19 |  | 0.069 | 0.037 | 0.526 | -0.145 | 0.284 |
| Stigmatization due to COVID-19 | 0.205 | 0.294 | 0.009 | 0.052 | 0.357 |  | 0.033 | 0.131 | 0.282 | -0.027 | 0.093 |
| Exposure to traumatic events | -0.083 | -0.011 | 0.729 | -0.551 | 0.386 |  | 0.078 | 0.03 | 0.41 | -0.107 | 0.263 |
| Witness of traumatic events | 0.23 | 0.033 | 0.262 | -0.172 | 0.632 |  | -0.065 | -0.025 | 0.424 | -0.224 | 0.094 |
| Social Support | -0.298 | -0.317 | <.001 | -0.374 | -0.223 |  | -0.138 | -0.406 | <.001 | -0.168 | -0.108 |
| COVID-19 Status | -4.423 | -0.076 | 0.129 | -10.136 | 1.289 |  | 0.34 | 0.016 | 0.767 | -1.916 | 2.597 |
| Gender*Exposure to COVID-19 | -0.373 | -0.112 | 0.168 | -0.902 | 0.157 |  | -0.304 | -0.252 | 0.004 | -0.513 | -0.095 |
| Gender*Exposure to COVID-19 | 0.008 | 0.001 | 0.981 | -0.696 | 0.713 |  | 0.088 | 0.039 | 0.534 | -0.19 | 0.366 |
| Gender*COVID-19 status | -6.207 | -0.077 | 0.124 | -14.124 | 1.71 |  | -3.98 | -0.137 | 0.013 | -7.108 | -0.853 |
| Gender*Stigma Ebola | -0.081 | -0.103 | 0.49 | -0.31 | 0.149 |  | -0.024 | -0.084 | 0.607 | -0.114 | 0.067 |
| Gender*Stigma COVID-19 | 0.039 | 0.051 | 0.693 | -0.157 | 0.236 |  | -0.006 | -0.022 | 0.879 | -0.084 | 0.072 |

Reference categories are the following: Sex: Men; Employment status: unemployed; Education level: university; Marital status: Single; participants ‘experiences: survivors; B: Unstandardized coefficients; Beta: standardized coefficients.
